# Enhancing capacities in genomic surveillance capabilities for SARS-CoV-2 and dengue virus: A South-South collaborative partnership

**DOI:** 10.1371/journal.pgph.0004365

**Published:** 2025-04-08

**Authors:** Sully Márquez, Gerald Vasquez-Aleman, Jose G. Juarez, Cristhiam Cerpas, Paul Cardenas, Shannon Bennett, Angel Balmaseda, Eva Harris, Josefina Coloma

**Affiliations:** 1 Universidad San Francisco de Quito, Quito, Ecuador; 2 Sustainable Sciences Institute, Managua, Nicaragua; 3 California Academy of Sciences, California, United State of America; 4 Laboratorio Nacional de Virología, Centro Nacional de Diagnóstico y Referencia, Ministerio de Salud, Managua, Nicaragua; 5 Division of Infectious Diseases and Vaccinology, School of Public Health, University of California, Berkeley, California, United State of America; Fundacao Oswaldo Cruz, BRAZIL

## Abstract

Latin American countries have faced limited access to new scientific technologies for many years due to restricted budgets for research programs, which has hindered local scientific development. These research disparities became especially evident during the COVID-19 pandemic, as lower-middle-income countries (LMICs) like Ecuador and Nicaragua had restricted access to genomic surveillance protocols, sequencing technologies, and adequate infrastructure, compromising global pandemic preparedness and response. In response to the urgent need for SARS-CoV-2 research capabilities in these countries, the Asian-American Center for Arbovirus Research and Enhanced Surveillance led the initiative, collaborating with the NGO Sustainable Sciences Institute and LMIC stakeholders, including universities and Ministries of Health, to develop pandemic-related research programs, provide resources, and conduct peer training workshops for local health scientists. Over the past five years, collaborative efforts have enabled teams in Ecuador and Nicaragua to establish sustainable research capacity and technology-sharing initiatives, as showcased by the institutionalization of government-led genomic surveillance efforts. This has opened new research opportunities in genomic surveillance for other emerging and reemerging pathogens and strengthening South-South collaboration.

## Introduction

In many Latin American countries, the national budgets assigned to research and development expressed as a percentage of the gross domestic product (GDP) are approximately one hundred-fold less than in high-income countries, where the research and development (R&D) investment is around 3.5% of the GDP [1,2]. As a result, these countries with little R&D investment are considered “scientifically lagging”, lacking the local capacity to use novel scientific technology and, more importantly, to establish successful research programs. In the field of biological and health sciences specifically, many investigators from developed nations have engaged in colonizing approaches to research for decades, considering Latin America as the source of prime material and samples to conduct their studies without scientists’ intellectual and active participation on the ground. There are, however, many instances of successful collaborations between high and low-income countries (LMICs), as well as South-South collaborations, that have been a motor for research advancement in resource-limited settings [3–5]. Programs that build and strengthen local capacities, enhance existing research infrastructure, and empower a science-based workforce are essential for long-term sustainability and truly collaborative science.

Although these programs are a lifeline for LMIC scientists and are funded mainly by the US government through its various research, health, or aid agencies (e.g., NIH, NSF, USAID, CDC, etc.) through collaborative contracts, sub-awards, and limited number of direct grants from global health foundations (e.g., Gates Foundation, Wellcome Trust, Doris Duke Charitable Foundation, Rockefeller Foundation, etc.), existing rules on administrative cost funding and grants management automatically exclude many institutions and even countries from participating. Indirect costs (IDC) rates are capped at amounts that do not reflect the actual costs of managing a grant or maintaining facilities. US-based institutions have negotiated indirect cost rates of 50% to over 90%, while in Latin America, the IDC is capped at 8% for most NIH grants and up to 10-15% for other funders [6–8]. Another major limitation of LMICs is that local government funding for research is low to nonexistent, with insufficiently small grants when local funding does exist. While labor costs are much lower than in the US or Europe, most reagents and supplies are imported with very high tariffs, resulting in prices up to four times those in the US for the same items [2].

This research inequality was evident by the unbalanced resource distribution for genomic and epidemiological surveillance of SARS-CoV-2, reflected partly by the number of genomes deposited in the GISAID database. About 350,000 sequences were uploaded by African and Central and South American countries, compared to 9.5 million uploaded by Europe and North America [9]. These numbers do not reflect the force of infection or intensity of the pandemic but rather the barriers to implementing genomic surveillance that were representative of what was happening on the ground. LMICs faced challenges such as establishing infrastructure and sequencing technologies, developing and adapting protocols with the inherent difficulty of acquiring the necessary tools and reagents in a competitive global market and maintaining and expanding this new approach. Through international collaborations with programs focused on capacity strengthening, genomic surveillance was implemented, leapfrogging the technological challenges to meet the needs of the extraordinary circumstances imposed by a global pandemic.

Implementing genomic surveillance programs has become essential to understanding the evolution and spread of infectious diseases (Box 1), especially in climate change and increased global mobility. This is particularly relevant in Latin America, where there has been a spike in transmission and dissemination of arboviruses such as dengue, Zika, chikungunya, and recently Oropouche and, more broadly, with the recent emergence and/or spread of diseases like COVID-19 and Mpox. Genomic studies shed light on the evolutionary paths of emerging pathogens and provide crucial information for developing prevention, diagnostic, and treatment strategies. The COVID-19 pandemic, despite its disastrous effects, allowed scientists in Latin America and around the world to apply sequencing and bioinformatic tools to understand the spread of variants in an unprecedented collaborative effort that enriched the collective knowledge of the evolution and transmission of SARS-CoV-2, which led to better decisions on vaccine modifications, regional and global travel advisories, and safety protocols.

**Box 1. Establishing global partners in emerging infectious diseases.**

The Centers for Research in Emerging Infectious Diseases (CREID; https://creid-network.org), a network established by the National Institutes of Health before the COVID-19 pandemic. CREID was designed to enhance research responses during outbreaks by prioritizing local capacity building. One of the funded centers, the Asian-American Center for Arbovirus Research and Enhanced Surveillance (A2CARES), led by Drs. Eva Harris and Josefina Coloma, focuses on infectious disease research, particularly arbovirus transmission, and includes collaborations with U.S. institutions (University of California (UC) Berkeley, University of Michigan, and others) and international partners in the United Kingdom (Imperial College London, the Sanger Institute, Oxford University, National Health Institutes), Nicaragua, Ecuador, and Sri Lanka. CREID’s emphasis on capacity enhancement, collaboration, peer training, and local agency was instrumental in establishing *in situ* genomic capabilities for the COVID-19 response and extending these efforts to other emerging diseases.

Two countries that illustrate the status of many scientifically lagging countries in Latin America pre-pandemic and the role of the academic sector in the research and genomic surveillance responses are Nicaragua and Ecuador. Here, we describe how this partnership evolved and the successful results of strengthening national surveillance programs.

## Scientific collaboration: The role of academia and civil society

**Fig 1:**
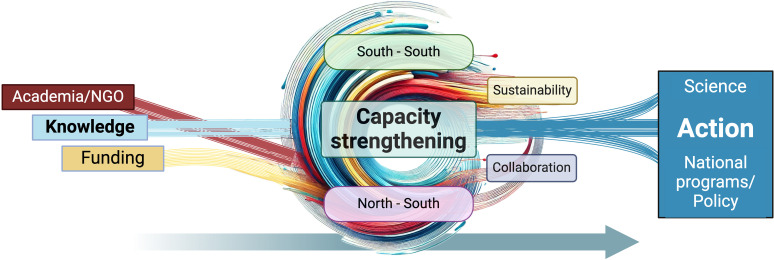
Conceptual diagram of the collaboration road map from academia to national programs/policy, with technology transfer and North-South and South-South capacity strengthening. This image was partially created using DALL·E (OpenAI).

Since the early 1990s, Nicaragua has been at the forefront of molecular and serological diagnostics for arbovirus surveillance in Latin America [10–19]. This has been promoted and enhanced through long-term scientific collaborations with an international US-based NGO (Sustainable Sciences Institute, SSI), whose mission is to support scientific and public health communities in resource-poor settings and develop sustainable local research and public health systems. SSI has also collaborated with Ecuador for a long time. SSI collaborates with partners across the globe to help them meet their public health needs locally. Through partnerships with researchers from academia, including its founders, SSI President (Harris), and Executive Director (Coloma), and funding from US institutions and global health foundations, SSI has over 30 years of long-standing collaborations with Ministries of Health and universities worldwide. Core to its mission, SSI has served as a capacity-strengthening and peer training platform for LMIC scientists. Its focus on transferring knowledge, expertise, and top-tier technology to low-income settings underscores how academia and nonprofits have a crucial role as repositories of knowledge and scientific proficiency (Fig 1).

Pre-pandemic, Nicaragua and Ecuador had distinct strengths in research, with familiar stakeholders such as SSI, UC Berkeley., and University of Michigan. Nicaragua excelled in developing in-house serological tools for arbovirus diagnosis. At the same time, Ecuador had advanced in sequencing methods and bioinformatics for studying microorganisms such as dengue virus (DENV) in the context of collaborative research led by US and local investigators. The COVID-19 pandemic triggered a collaborative learning path between these countries. Their inclusion in A2CARES (Box 2) facilitated significant capacity-building and technology transfer initiatives, addressing pandemic-related research needs and strengthening scientific research in both nations. Post-pandemic, both countries have enhanced capacity development, cooperation, and technology transfer competencies and have acquired proficiency in critical new methodologies. Genomic surveillance remains a key focus of A2CARES, alongside promoting innovative technologies, including metagenomics, for future outbreak preparedness in a North-South collaboration. Efforts are also directed at local knowledge dissemination, exemplified by SSI Nicaragua’s initiatives to equip its Ministry of Health with genomic surveillance techniques. Here, we present the experiences, strategies, and lessons learned from the capacity sharing and strengthening activities between Nicaragua and Ecuador and beyond within the A2CARES framework and how the newly acquired capacities regarding SARS-CoV-2 propelled leveraging of new research opportunities in genomic surveillance for DENV and other emerging pathogens (MPox, Oropouche, etc.), with further peer technology transfer for serological assays.

**Box 2. Asian-American Center for Arbovirus Research and Enhanced Surveillance (A2CARES).**

One key partner that illustrates this multidirectional relationship is A2CARES, one of the ten CREID Centers globally. Based out of the University of California, Berkeley, and led by Drs. Harris and Coloma, A2CARES is a consortium of world-renowned investigators in arbovirology, epidemiology, immunology, diagnostics, phylogenetics, clinical research, and One Health. A2CARES developed an interconnected, harmonized network of clinical and laboratory sites to build research programs, compare epidemiology across regions, develop and implement cutting-edge diagnostic methods, and strengthen local and national capacity to respond efficiently and effectively to outbreaks. Standardized hospital studies and harmonized community-based cohorts in Ecuador, Nicaragua, and Sri Lanka have served as a basis to characterize and compare emerging infectious diseases across an urbanicity spectrum. With a focus on arthropod-borne viruses, A2CARES developed new molecular and serological tools and innovative research approaches to study transmission dynamics and risk factors for emergence and outbreaks. Although A2CARES was mainly set up to study arboviruses, COVID forced a strong pivot to support research and surveillance of SARS-CoV-2 in the three participating countries. The A2CARES PIs mobilized to first implement RT-PCR in-country at the NGO (SSI) and partner Universities. They also facilitated the transfer of thousands of serological assays to detect infection (pre-vaccines) and enabled the South-South exchange of expertise in genomic sequencing and serological assays, as described below.

## Landscape of sequencing capabilities at Universidad San Francisco de Quito-Ecuador and SSI-Nicaragua before the COVID-19 pandemic

Before the COVID-19 pandemic, Universidad San Francisco de Quito (USFQ)-Ecuador and SSI-Nicaragua commonly used conventional and real-time PCR techniques for molecular diagnosis and surveillance. For whole genome sequencing, metagenomics, and amplicon sequencing, samples and PCR products were sent abroad to international collaborators in the North (USFQ: Public Health England; SSI-Nicaragua: Broad Institute and others) or commercial providers such as Macrogen (South Korea). Implementing next-generation sequencing (NGS) *in situ* seemed unattainable due to several factors, including the lack of specific skills and expertise among laboratory personnel, the need for specialized laboratory facilities and new equipment, and the high cost of the technology. Efforts to establish genomic sequencing *in situ* started at USFQ in mid-2019 (pre-pandemic) and in Nicaragua in mid-2021 (during the pandemic).

USFQ obtained an initial collaborative grant to conduct the first *in situ* metagenomic sequencing study in Ecuador using Nanopore technology, focusing on arboviruses. Collaborators at Public Health England provided training to the USFQ team through a two-week qRT-PCR and metagenomic sequencing course for arboviruses (Mayaro, Oropouche, Zika, chikungunya, dengue) and other pathogens. In SSI-Nicaragua, all genomic sequencing before the COVID-19 pandemic until July 2021 was sent abroad to collaborators for processing and analysis.

## The start of genomic surveillance

Neither country was ready for what was to come and how fast SARS-CoV-2 would spread after a novel global coronavirus raised alarms about a potential pandemic in late 2019. Academic collaborators at UC Berkeley realized the impending threat. They obtained primers, protocols, and positive controls early on, enabling Nicaragua and Ecuador to have in-country capacity for molecular detection of SARS-CoV-2 by early February 2020. The first cases of COVID-19 were reported in Ecuador and Nicaragua by February and March of 2020, respectively, even before a pandemic was officially declared [20]. This novel health threat underscored the urgent need for *in situ* genomic surveillance to detect variants and understand the circulating lineages’ genomic epidemiology [21,22]. As such, at the USFQ, the initial project for genomic surveillance shifted from arboviruses to tracking SARS-CoV-2. Whole-genome sequencing was implemented using the Primer Scheme (V1 and V3) developed by the ARTIC network for nCoV-2019 using Nanopore technology, provided by collaborators from Oxford University.

### Peer training and capacity enhancement

In April 2021, via A2CARES, the team at USFQ, which had successfully implemented the ARTIC sequencing protocol in Quito, prepared and delivered online workshops for partners in Nicaragua, Ecuador and Paraguay (freely available course at https://www.youtube.com/watch?v=oLAWRp9USCY). The workshop sessions included theoretical presentations, recorded videos, and access to cloud repositories with academic resources and work protocols, ensuring that all participants had free access to these online resources. As sequencing capacity was established, both USFQ and SSI-Nicaragua could carry out genomic surveillance of SARS-CoV-2 variants of concern (VOCs). Several lineages were identified that circulated in both countries, including Alpha (January 2021), Gamma (March 2021), Delta (June 2021), and Omicron [23]. Collaborators at the California Academy of Sciences conducted additional in-person training in Nicaragua. As a direct result of the training, workshops, and funding provided by A2CARES, *in situ* implementation of genomic sequencing was established in SSI-Nicaragua and USFQ, which then served as the national genomic surveillance systems during the peak of the COVID-19 pandemic, with the national systems also being supported by PAHO. Through December 2023, these efforts resulted in 2,659 SARS-CoV-2 sequences generated by USFQ and 1,208 by SSI-Nicaragua. All sequences were uploaded to the GISAID database in a timely manner.

## Leveraging SARS-CoV-2 sequencing knowledge for dengue virus and beyond

Dengue is a vector-borne disease transmitted by mosquitoes of the *Aedes* genus, with the highest occurrence in tropical and subtropical regions. Dengue, endemic in over 130 countries, with hundreds of millions of infections worldwide, is one of the most critical global public health threats [24]. SSI has been at the forefront of dengue surveillance [10,11,13–19,25–27] and control [28,29] in Latin America, providing tools to empower local researchers to establish diagnostics at low cost [30–32] and help shape public health policy locally [33,34] and internationally [35–37]. Building local agency, long-term empowerment, and sustainability goes beyond sharing technology and protocols. This is exemplified by the commitment from SSI and A2CARES to develop local agencies. It requires commitment from academic researchers in Northern countries to invest in the personal and professional growth of LMIC team members, viewing them as true collaborators with scientific potential rather than cheap labor to ensure their academic success. These engagement efforts can ripple effect via additional peer training within LMICs, potentiating capacity strengthening of local personnel in scientific collaboration and research projects.

In 2021, USFQ reactivated arbovirus surveillance in Northern Ecuador (Esmeraldas province) as part of the A2CARES cohort study to generate full-length DENV genomic sequences. Collaborators at Oxford University shared the primer schemes and protocol for DENV sequencing, enabling USFQ to generate 27 complete DENV genomes for DENV-1 and DENV-2 [38]. From this initial effort in Ecuador, an online workshop on DENV phylogenetics and phylodynamics, led by teams from USFQ, California Academy of Sciences, and Oxford University and facilitated by A2CARES, was carried out with participants from Ecuador, SSI-Nicaragua, and Paraguay in April of 2021. This workshop addressed topics related to the basic principles of evolutionary theory, fundamentals of phylogenetic analysis, and construction of maximum likelihood trees, which enabled the teams to conduct their phylogenetic analysis *in situ*. Like the previous SARS-CoV-2 workshop, educational materials were shared on the cloud, providing free access to all participants (see data sharing section). Following this successful workshop, SSI-Nicaragua organized an on-site dengue sequencing training session through SSI and A2CARES, led by the USFQ team from Ecuador from July 10-23, 2022. During this same visit, the SSI-Nicaragua team conducted reciprocal training for the USFQ team on serological assays for DENV and SARS-CoV-2. These hands-on workshops are a best-practice example of the benefits of peer training and collaboration efforts between LMIC scientists, coupled with additional hands-on training in bioinformatics and phylogenetics by partners at the California Academy of Sciences. As a direct result, 113 DENV full-length genomic sequences were produced in SSI-Nicaragua, and a total of 60,000 serological tests were s performed by using the NS1 BOB-ELISA, Inhibition ELISA and capture ELISA assays in Ecuador at the Universidad Central del Ecuador, another A2CARES partner where serological assays are implemented.

Beyond our North-South and South-South collaborative efforts in genomic surveillance, over the past 5 years, the teams from Nicaragua and Ecuador have engendered a longstanding capacity and technology-sharing collaboration within the framework of A2CARES/CREID, in which the research sites are in constant communication for troubleshooting, manuscript-writing support, and new project collaborations on other topics. Additionally, we are harmonizing data and laboratory Standard Operating Procedure (SOP) among all the A2CARES research sites in Nicaragua, Ecuador, and Sri Lanka, showcasing our strong collaboration through a multi-stakeholder endeavor. Through SSI and A2CARES partners in the US (Emory University, University of North Carolina-Chapel Hill), serological and molecular protocols and reagents have been shared with SSI-Nicaragua, the Universidad Central of Ecuador, USFQ, Instituto Nacional de Investigación en Salud Pública of Ecuador (INSPI Ministry of Health of Ecuador), and the University of Jayewardenepura (Sri Lanka). Additionally, with the support from A2CARES, researchers from SSI-Nicaragua, USFQ, INSPI, and the University of Jayewardenepura participated in metagenomic courses at Columbia University for pathogen detection in undiagnosed febrile illness serum samples using the VirCap-Seq method [39], furthering the genomic surveillance capacities in all countries.

More recently, in August 2024, a collaborative project regarding the genomic surveillance of rabies in Nicaragua was undertaken by members of SSI-Nicaragua and USFQ. This collaboration, extending beyond the efforts of A2CARES, has led to the implementation of molecular diagnostics for rabies virus and whole-genome sequencing of 9 rabies virus genomes thus far. This project aims to assist Nicaragua in achieving certification for country or zone free from dog-mediated rabies. The future of this South-South collaboration is indeed promising, to continue these training projects and empower Nicaraguan and Ecuadorian scientists to use and implement sequencing technologies for a deeper understanding of endemic, emerging, and re-emerging infectious diseases. Ecuador has already implemented genomic surveillance for the avian flu (H5N1), respiratory syncytial, and pox. The ultimate goal is to share this knowledge with other critical stakeholders within Latin America and beyond.

## Sharing capabilities beyond A2CARES

The USFQ team has organized several workshops to train fellow scientists in implementing sequencing capacity locally and in other countries. The first was designed in collaboration with the Welcome Connecting Science organization and was a virtual workshop entitled “SARS-CoV-2 Bioinformatics for Beginners”, which took place from October 31 to December 2, 2022. Another workshop was designed in collaboration with Jhpiego-Ecuador for national laboratories from Guatemala, Ghana, and Cameroon and took place virtually and on-site in December 2023. The virtual section contained a theoretical component about bioinformatic pipelines with the Linux Platform and its applications using Oxford Nanopore Technology (ONT), and the on-site part consisted of library preparation and sequencing using this technology. Moreover, in December 2023, due to the avian influenza outbreak, the USFQ team trained personnel from Agrocalidad, Ecuador, to perform sequencing in their laboratory.

In Nicaragua, SSI organized workshops to continue capacity building across Nicaragua. In February 2023, a series of workshops were held with Ministry of Health personnel to transfer knowledge about genomic sequencing. Participants from the National Center for Diagnosis and Reference in areas such as Water and Food Microbiology, Bacteriology, Mycobacteria, Entomology, and Virology attended. The workshop program included content on general principles of molecular biology, sequencing methods, library preparation, basic concepts of computational methods applied to genomic data, and pipelines for genome assembly.

## Final thoughts

Developing local human resources is a complex, time-intensive, and costly endeavor. Within SSI and A2CARES (global North partners), a strong emphasis is placed on fostering regional capacity building, empowering researchers from LMICs and making data FAIR (Box 3). Our results demonstrate the benefits of a global multistakeholder collaboration and show how local teams in low-income countries can successfully decentralize top-tier technology when in-country capacity is a critical element of research objectives and a core focus of the lead PI’s. These efforts have enabled Nicaragua and Ecuador to establish their own national genomic surveillance efforts, with further funding and support from international and national organizations such as PAHO and CDC. Additionally, sharing data and analyses enables a deeper and broader understanding of infectious disease dynamics at a regional level, significantly contributing to public health in Latin America and beyond.

**Box 3. Making data FAIR (Findable, Accessible, Interoperable and Reusable).**

The new requirement from the NIH for data management and sharing policy (Findable, Accessible, Interoperable, and Reusable; FAIR), effective since January 2023, underscores the importance that research funded by the NIH develops a plan for managing and sharing scientific data. The CREID network has embraced this to ensure all Research Centers can share their data as efficiently and quickly as possible, not only within the CREID network but around the globe. We aim to make our sequences freely available to the Latin American scientific community and beyond. Full-length SARS-CoV-2 genomic sequences generated in Ecuador and Nicaragua were uploaded to the GISAID platform (https://gisaid.org/) with associated metadata such as sample collection date, source, patient status, and technology used. DENV genomic sequences were uploaded to the GENBANK database with their corresponding accession number; in addition, Ecuadorian DENV sequences were also uploaded to the EpiArbo database, a new data-sharing platform built on the success of the GISAID EpiFlu and EpiCov databases. The commitment of NIH and CREID to open data showcases the importance of how real-time information can help guide public health policy and manage disease outbreaks in the most effective way possible.
